# The impact of pre-ictal statin use on vasospasm and outcome in aneurysmal subarachnoid hemorrhage

**DOI:** 10.1007/s00701-023-05812-2

**Published:** 2023-10-04

**Authors:** S. Andersen, E. Western, W. Sorteberg, A. Sorteberg

**Affiliations:** 1 Institute of Clinical Medicine, University of Oslo, P.B. 1072, 0316 Blindern, Oslo Norway; 2 Department of Physical Medicine and Rehabilitation, Oslo University Hospital, Nydalen, P.B. 4950, 0424 Oslo, Norway; 3 Department of Neurosurgery, Oslo University Hospital, Nydalen, P.B. 0454, 0424 Oslo, Norway

**Keywords:** Aneurismal subarachnoid hemorrhage, Statins, Vasospasm, Outcome, Fatigue

## Abstract

**Background:**

Pleiotropic effects of statins may be beneficial in alleviating cerebral vasospasm (VS) and improving outcome after aneurysmal subarachnoid hemorrhage (aSAH). Initiation of statin treatment at aSAH is not recommended; however, the effect of pre-ictal and continued statin use is not fully investigated.

**Methods:**

Retrospective study comparing aSAH patients admitted in 2012 to 2021 with pre-ictal statin use versus those not using statins. Patient entry variables, radiological/sonological VS, symptomatic VS, and radiologically documented delayed cerebral ischemia (DCI) were registered. Outcome was scored in terms of mortality, modified Rankin score, Glasgow outcome score extended, and levels of fatigue. Patients were compared on group level and in a case–control design.

**Results:**

We included 961 patients, with 204 (21.2%) statin users. Statin users were older and had more often hypertension. Severe radiological/sonological VS, symptomatic VS, and DCI were less frequent in statin users, and their length of stay was shorter. Mortality, functional outcome, and levels of fatigue were similar in both groups. When analyzing 89 pairs of statin users and non-statin users matched for age, aSAH severity, gender, and hypertension, we confirmed decreased radiological/sonological and symptomatic VS as well as shorter length of stay in statin users. They also had more often a favorable functional outcome and lower levels of fatigue.

**Conclusions:**

Patients with pre-ictal and continued use of statins have a reduced occurrence of radiological/sonological and symptomatic VS, shorter length of stay, and more often favorable functional outcome, whereas mortality is similar to non-statin users. Even though larger multicenter studies with common, strict protocols for prevention, diagnosis, and treatment of vasospasm are needed to finally establish the value of statins in aSAH, continuation of pre-ictal statin use seems worthwhile.

## Introduction

Vasospasm (VS) after aneurysmal subarachnoid hemorrhage (aSAH) refers to a transient narrowing of the large cerebral arteries and may lead to delayed cerebral ischemia (DCI). Vasospasm after SAH is common and diagnosed in up to 70% on angiography, whereas symptomatic VS with neurologic deterioration occurs in about 30% [9]. Vasospasm is a feared complication to aSAH and is associated with poorer outcome [25]. The pathophysiology of VS is a complex cascade of events that is not yet fully understood. The amount of intraventricular blood (IVH) and thickness of SAH seem to be correlated with the risk of developing VS [16]. It is also argued that inflammation, interruption in the endothelial metabolism (reduced nitrogen oxide (NO) availability), secretion of endothelin-1, and oxidative stress can cause VS [2, 11, 39]. Hitherto, nimodipine is the sole recommended substance in preventing DCI as a result of VS, as it has been shown to improve neurological outcome after aSAH [22].

Due to their pleiotropic effect, it has been investigated whether statins can reduce VS. The main effect of statins is to inhibit HMG-CoA-reductase, which will reduce the amount of LDL-cholesterol. Statins increase the expression and activity of endothelial NO synthase, which will lead to more NO availability [20]. NO is a potent vasodilator, neuroprotector, and promotor of angiogenesis. It also protects the endothelium from platelet adhesion, which is thought to reduce cell apoptosis. Other studies have shown that statins can stabilize the endothelial cells and preserve their function on NO availability, lower inflammation, and reduce brain edema [31, 32]. These mechanisms are thought to be beneficial against VS and DCI and may further better outcome. Only lipophilic statins like atorvastatin and simvastatin pass the intact blood–brain barrier [54], and it hence remains unclear if hydrophilic statins exert similar pleiotropic effects on the brain.

Several randomized controlled trials (RCTs) have studied the effect of initiating statin treatment at admission in aSAH [27, 49, 50]. Some trials administered the hydrophilic statin pravastatin, whereas others used the lipophilic statin simvastatin [49]. The largest multicenter RCT “STASH” found no benefit of simvastatin administered at admission [27]. A meta-analysis of RTCs from 2019 concluded that statins reduce VS, DCI, and mortality in aSAH [49]. A meta-analysis from 2017 that included 1121 of the 1597 cases in the aforementioned meta-analysis could, however, not find any benefit of statins regarding symptomatic VS, DCI, or mortality [48]. This discrepancy may be due to the heterogeneity of the included studies with regard to the type and dose of statins administered and their lack/variety of VS definitions. Consequently, current guidelines do not recommend the initiation of statins upon aSAH but do not address the continuation of pre-ictal statin use [22]. This is a question that needs to be answered by retrospective studies with sufficient power.

Preload with statins, i.e., pre-ictal statin use may have a different effect on the development of VS and outcome than initiation of statins at ictus as some studies found that the length and dose of administration were crucial for the effect of statins [52]. Some studies have investigated the effect of pre-ictal statin use on VS and outcome [36, 40]; however, these were underpowered with regard to the number of statin users (all less than 50 patients) and hence failed to detect a significant impact on VS or outcome. Moskowitz et al. [40] found a trend towards less VS among statin users but included only 26 statin users and scored both symptomatic, radiological, and sonological VS as one entity. Lizza et al. [36] found no differences in functional outcome after aSAH in statin users versus non-statin users, and all their 41 patients used the hydrophilic pravastatin. Nowadays, the lipophilic atorvastatin is the most prescribed statin worldwide [35], but its effect in aSAH remains understudied.

So far, outcome has been studied in terms of mortality and functional status. Functional status is highly influenced by fatigue [4], which is a common and often permanent sequel after aSAH [29]. The pathophysiology of chronic post-aSAH fatigue has not yet been delineated, though it has been hypothesized that post-aSAH fatigue is due to inflammation causing a dopamine imbalance [13, 24]. Inflammation may hence be a common denominator for the development of both VS and fatigue. Given that there is an anti-inflammatory effect of statins, they may have a potential positive impact on both the development of VS and on the extent of post-aSAH fatigue. No studies have hitherto investigated this topic.

The aim of the present study was to retrospectively compare the frequency and severity of VS, outcome, and levels of post-aSAH fatigue in a larger number of aSAH patients with pre-ictal and continued statin use (prevailing atorvastatin). We also want to introduce dose/effect relationships of statins since no former studies within aSAH have included this aspect. To this end, we compared statin users with non-statin users on group level and with a case–control design.

## Materials and methods

### Patients

All patients admitted to the Department of Neurosurgery, Oslo University Hospital, Oslo, Norway, with aSAH are registered in an internal quality registry approved by the data protection officer (11/6692). For the present study, data were retrieved for patients admitted during 2012 throughout 2021 after approval as a quality project by the data protection officer in accordance with the Patient Journal Act §6 and Health Personnel Act §26 (project approval 21/10232); signed consent was waived due to the nature of the study.

### Institutional treatment principles

Our treatment algorithm has previously been described [51]. All patients are under continuous clinical surveillance in the general intensive care unit or the neurointermediate ward if not dependent on invasive mechanical respiratory support. We routinely perform cerebral computed tomographic angiography (CTA) to radiologically diagnose VS, on day 7 in awake and on day 5 in sedated and intubated patients, and thereafter when needed. Transcranial Doppler (TCD) is also performed frequently to sonologically detect and follow the development of VS. All patients receive intra-venous (i.v.) nimodipine upon arrival, initiated at 15 μg/kg/h and increased to 30 μg/kg/h when no drop in blood pressure is observed. After aneurysm repair and if the patient can swallow, nimodipine is administered orally with 60 mg × 6 for a total length of 21 days. We aim at maintaining normovolemia and the cerebral perfusion pressure (CPP) above 70 mmHg. In cases with severe and/or symptomatic VS, the CPP lower limit is elevated to 90 mmHg. If the symptoms from VS do not resolve upon increasing the CPP, patients receive rescue therapy with intra-arterial (i.a.) nimodipine until VS resolves.

### Variables

Statin users were defined as patients being treated with statins prior to the ictus and who continued their statin dosage while hospitalized. Non-statin users were defined as patients not being treated with statins.

The following data were registered: age, gender, and previous medical history. Clinical status prior to aneurysm repair and prior to intubation was expressed with the World Federation of Neurosurgical Societies (WFNS) grading system [45]. From the first available CT scan, we scored the amount of SAH using the Fisher score [14], the presence and size of intracerebral hematomas (ICH), and acute subdural hematomas (ASDH); for the amount of IVH, we used a modified LeRoux score where 0 means no IVH [33]. We measured the amount of midline shift and registered aneurysm location, size, and multiplicity.

We also registered mode of aneurysm repair; if both surgical and endovascular were performed, we registered as surgical. We noted if tracheostomy, hemicraniectomy, or a cerebrospinal fluid (CSF) shunt were performed. The length of stay was noted.

Vasospasm was divided into vessel narrowing as diagnosed radiological/sonological (CTA or TCD; the highest degree from either method was scored). We categorized VS as follows: none, up to moderate in 1 vessel, up to moderate in multiple vessels, severe in 1 vessel, and severe in multiple vessels. Symptomatic VS was defined as any delayed neurological deterioration that could not be attributed to rebleeding, hydrocephalus, intracerebral hematoma, electrolyte abnormalities, or toxic and metabolic factors, including respiratory abnormalities and infection [17]. DCI refers to any radiologically visible ischemic lesion not caused by the hemorrhage or by aneurysm repair.

Date of last follow-up for mortality was November 4, 2022, and mortality, regardless of cause, was scored at 30 days and 1 year. Functional outcome was expressed with the modified Rankin scale [3] and the Glasgow outcome scale extended [59]. Post-aSAH fatigue was expressed using the fatigue severity scale (FSS) mean score [28], where a mean FSS ≥ 4 indicates clinically significant fatigue.

### Statistics

Statistical analysis was performed in SPSS v 28.0.1.1 (IBM SPSS statistics for Windows and Macintosh v.28.0.1.1, Armonk, NY). Categorical variables are presented as frequencies or percentages, whereas continuous variables are presented by mean and range if normal distributed or median and interquartile range if not normal distributed. Statin users and non-statin users were compared as independent groups using the chi-square test (categorical data), T-test (normal distribution), or Mann–Whitney U test (not normal distribution). Uni- and multivariate analyses were performed to identify possible predictors of VS and DCI. Patients living long enough to develop VS after the ictus (day 5) were included in this subanalysis. Any variable with *p* < 0.100 in the univariate analysis was included in the multivariable model, unless there occurred collinearity. For VS and outcome data, we also matched pairs of statin users and non-statin users by age (fuzzy factor 5.0), exact match of Fisher and WFNS grade, and by hypertension (yes/no). When there was more than one possible match, we chose the match with the same gender. A significance level of 5% was adopted, and all *p*-values are provided for 2-sided tests.

## Results

### Patients, admission, and treatment data

A total of 961 patients were included in the study with 204 (21.2%) pre-ictal statin users among them. Most statin users had been prescribed atorvastatin (53.2%), whereas 36.8% took simvastatin, 6.0% rosuvastatin, 2.5% pravastatin, and 1.5% fluvastatin. Table 1 presents patient characteristics at admittance for statin users and non-statin users. Statin users were on average 12 years older and had significantly more often hypertension. Non-statin users presented more often in WFNS grade 5 but less often in WFNS grade 3. Statin users had a shorter length of stay. We could match 89 pairs of statin users to non-statin users.

**Table 1:** Patient characteristics and treatment data

	Statin user	Non-statin user	*p*-value
Total number = 961	*n* = 204 (21.2%)	*n* = 757	
Age	*67.2* ± *9.7*	*54.95* ± *14.5*	< *0.001*
Female (%)	61.3	65.5	0.260
Hypertension (%)	*74.3*	*30.3*	< *0.001*
Smoking status (%)	51.4	57.7	0.127
Never	24.0	25.8	0.636
Earlier	*19.6*	*10.7*	< *0.001*
Current smoker	46.1	49.5	0.093
Unknown	10.3	14.2	0.092
WFNS grade (%)			
1	35.8	31.2	0.418
2	21.1	24.6	0.390
3	*12.3*	*6.9*	*0.007*
4	17.6	15.6	0.343
5	*13.2*	*21.8*	*0.007*
Intraparenchymal hematoma (%)			
None	69.2	64.0	0.217
< 2 cm	13.8	12.5	0.653
2–5 cm	9.4	14.4	0.100
> 5 cm	7.5	9.0	0.568
Acute subdural hematoma (%)	10.3	6.5	0.060
LeRoux score [33] (median, IQR)	3.0 (0.0, 7.0)	3.0 (1.0, 6.0)	0.890
Rebleed prior to repair (%)	11.3	9.5	0.510
Aneurysm size (mm)	7.65 ± 5.48	7.44 ± 5.68	0.339
Multiplicity (%)	27.5	26.0	0.669
No aneurism repair (%)	10.3	7.9	0.303
Surgical repair (%)	40.6	45.6	0.389
Endovascular repair (%)	49.0	48.2	0.797
Hemicraniectomy (%)	2.0	2.1	0.930
Tracheostomy (%)	19.6	25.8	0.099
Cerebrospinal fluid shunt (%)	28.9	26.0	0.213
Length of stay (days)	*12.8 (6.8, 18.2)*	*15.2 (8.5, 20.0)*	*0.012*
Time from ictus to arrival (hours)	4.8 (2.8, 16.7)	5.2 (3.2, 23.3)	0.448

Significant difference in italics

*WFNS* World Federation of Neurosurgical Societies [45], *IQR* interquartile range

### Vasospasm

**Fig. 1:**
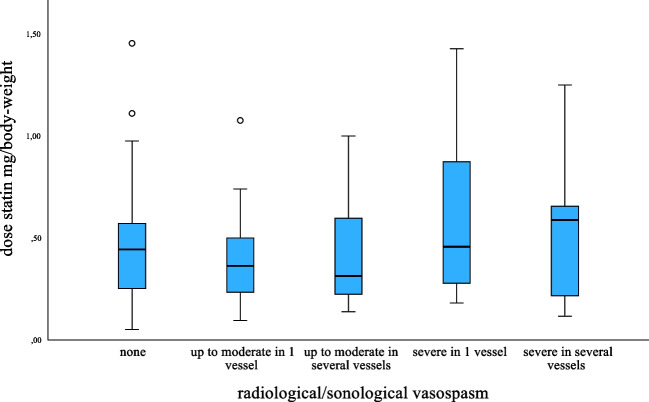
Statin dose (mg/kg body weight) versus vasospasm category

Table 2 shows that radiological/sonological VS was less common in statin users. They also had significantly less frequent severe VS, symptomatic VS, and DCI. The non-statin users were more often selected for rescue therapy with i.a. application of nimodipine. The statin dose/kg body weight was not linked to the occurrence or severity of VS (Fig. 1, *p* = 0.538).

**Table 2:** Cerebral vasospasm in statin users and non-statin users

	Statin user	Non-statin user	*p*-value
Vasospasm upon arrival (%)	1.6	4.5	0.071
Time from ictus to arrival (hours)	4.8 (2.8, 16.7)	5.2 (3.2, 23.3)	0.448
Vasospasm (CTA-TCD) (%)			
*None*	*58.5*	*41.3*	< *0.001*
*Up to moderate in 1 vessel*	17.8	18.1	0.926
*Up to moderate in multiple vessels*	16.7	20.8	0.212
*Severe in 1 vessel*	4.4	8.3	0.080
*Severe in multiple vessels*	*2.8*	*11.5*	< *0.001*
Symptomatic vasospasm (%)	*9.8*	*21.8*	< *0.001*
Treated with i.a. nimodipine	*3.3*	*10.2*	*0.003*
Number of i.a. nimodipine treatments	2.5 (1.00, 3.75)	2.0 (1.0, 3.0)	0.413
Total amount i.a. nimodipine injected (mg)	10.5 (2.75, 22.50)	9.0 (3.0, 17.0)	0.969
Radiological DCI (%)	*9.9*	*17.2*	*0.017*

Significant differences in italics

*i.a.* intra-arterial, *DCI* delayed cerebral ischemia, *CTA* computed tomography angiography, *TCD* transcranial Doppler ultrasonography

Table 3 shows the uni- and multivariate analyses for predictors of VS and DCI (81 patients were excluded because they had died within 5 days after the ictus). Higher age (OR 0.0973, 95% CI 0.958–0.986) and statin use (0.494, 95% CI 0.260–0.939) were highly significant protective predictors of symptomatic VS. Statin use was not an independent predictor of radiological/sonological VS, severe VS, or DCI. Fisher grades 3 and 4 more than tripled the chance to develop radiological/sonological and symptomatic VS. The matched pair analysis confirmed that statin users more often had no VS and less radiological/sonological severe VS as well as symptomatic VS and approximately a 3-day shorter length of stay (Table 4). The frequency of DCI was similar in statin users and non-statin users.

**Table 3:** Predictors of symptomatic and severe vasospasm and delayed cerebral ischemia. Uni- and multivariate analyses

	Symptomatic vasospasm				Severe vasospasm (radiological/sonological)				Delayed cerebral ischemia			
	Univariate analysis		Multivariate analysis		Univariate analysis		Multivariate analysis		Univariate analysis		Multivariate analysis	
	Odds ratio (95% CI)	*p*-value	Odds ratio (95% CI)	*p*-value	Odds ratio (95% CI)	*p*-value	Odds ratio (95% CI)	*p*-value	Odds ratio (95% CI)	*p*-value	Odds ratio (95% CI)	*p*-value
Statin use	0.397 (0.236–0.668)	** < 0.001**	0.494 (0.260–0.939)	0.031	0.319 (0.176–0.579)	** < 0.001**			0.549 (0.324–0.929)	**0.026**		
Hypertension	0.688 (0.477–0.993)	**0.045**			0.744 (0.506–1.093)	0.131			0.771 (0.516–1.150)	0.203		
Age	0.976 (0.964–0.987)	** < 0.001**	0.972 (0.958–0.986)	< 0.001	0.961 (0.948–0.973)	** < 0.001**	0.956 (0.942–0.970)	< 0.001	0.995 (0.982–1.008)	0.457		
Current smoking	1.406 (0.979–2.020)	**0.065**			1.185 (0.815–1.724)	0.374			1.250 (0.822–1.901)	0.297		
Female	0.790 (0.559–1.118)	0.183			0.084 (0.679–1.424)	0.930			0.582 (0.401–0.846)	**0.005**	0.495 (0.321–0.764)	0.002
Body weight	1.003(0.994–1.013)	0.521			1.004 (0.994–1.014)	0.429			1.009 (0.999–1.020)	**0.088**		
Fisher > 2 [14]	2.921 (1.925–4.431)	** < 0.001**	3.240 (2.067–5.078)	< 0.001	2.431 (1.586–3.726)	** < 0.001**	3.273 (2.053–5.217)	< 0.001	2.974 (1.878–4.708)	** < 0.001**		
LeRoux score [33]	0.998 (0.958–1.040)	0.934			1.007 (0.965–1.050)	0.763			1.094 (1.051–1.138)	** < 0.001**		
Rebleed	0.859 (0.423–1.741)	0.672			1.283 (0.659–2.497)	0.463			3.449 (1.936–6.143)	** < 0.001**	2.137 (1.121–4.072)	0.021
Multiple aneurism	0.752 (0.503–1.123)	0.163			0.634 (0.409–0.982)	**0.041**			1.009 (0.974–1.046)	0.623		
Aneurism size	0.955 (0.915–0.988)	**0.038**			0.952 (0.909–0.997)	**0.036**			1.009 (0.974–1.046)	0.623		
Midline shift	1.010 (0.954–1.071)	0.723			1.046 (0.989–1.105)	0.113			1.027 (0.968–1.090)	0.380		
Acute subdural hematoma	1.336 (0.664–2.689)	0.417			1.188 (0.561–2.518)	0.653			2.481 (1.289–4.775)	**0.007**		
hemicraniectomy	1.959 (0.733–5.234)	0.180			2.938 (1.136–7.597)	**0.026**			2.579 (0.961–6.911)	**0.060**		
Aneurism repair												
Endovascular	Reference	**–-**			Reference	**–-**			Reference	**–-**		
Surgical	1.150 (0.819–1.616)	0.419			1.125 (0.786–1.608)	0.520			0.898 (0.619–1.302)	0.571		
None	0.788 (0.089–6.291)	0.788			2.057 (0.391–10.816)	0.394			1.296 (0.143–11.773)	0.818		
Intracerebral hematoma												
None	Reference	–-			Reference	–-			Reference	–-		
Less than 2 cm	1.195 (0.708–2.018)	0.505			1.450 (0.853–2.465)	0.169			1.762 (1.020–3.045)	**0.042**		
2–5 cm	1.787 (1.122–2.844)	**0.014**			1.441 (0.865–2.401)	0.161			2.523 (1.547–4.114)	** < 0.001**		
Over 5 cm	0.519 (0.217–1.238)	0.139			1.146 (0.559–2.349)	0.709			1.278 (0.603–2.709)	0.521		
Aneurism location												
MCA/ICA	reference	–-			Reference	–-			Reference	–-		
ACA/ACoA	0.916 (0.616–1.364)	0.667			1.123 (0.734–1.718)	0.593			1.327 (0.840–2.097)	0.225		
Vertebrobasilar	0.644 (0.409–1.013)	**0.057**			0.806 (0.498–1.305)	0.381			1.083 (0.656–1.790)	0.755		
WFNS grade [45]												
1	Reference	–-			Reference	–-			Reference	–-	Reference	–-
2	1.867 (1.189–2.931)	**0.007**			1.102 (0.677–1.794)	0.696			1.716 (0.945–3.114)	**0.076**		
3	1.350 (0.683–2.669)	0.388			1.081 (0.527–2.216)	0.832			3.033 (1.468–6.267)	**0.003**		
4	1.753 (1.049–2.931)	**0.032**			1.773 (1.066–2.948)	**0.027**			4.750 (2.683–8.408)	** < 0.001**	4.412(2.175–8.949)	< 0.001
5	2.184 (1.289–3.701)	**0.004**			1.828 (1.068–3.129)	**0.028**			5.269 (2.859–9.345)	** < 0.001**	3.869 (1.855–8.069)	< 0.001

Values with *p*-values written in bold font were included in the multivariate analysis. Results for the variables that reached a significant level in the multivariate analysis were included in the table

*ACA anterior cerebral artery, ACoA anterior communicating artery, MCA* middle cerebral artery, *ICA* internal carotid artery, *WFNS* World Federation of Neurosurgical Societies [45]

**Table 4:** Matched pair analysis

	Statin user	Non-statin user	*p*-value
	*n* = 89	*n* = 89	
Age (mean, ± SD)	66.5 ± 10.1	66.3 ± 10.3	0.906
Hypertension (%)	68.5		
Female (%)	60.7	61.8	0.878
WFNS grade [45] (%)			
1	46.1		
2	20.2		
3	9.0		
4	12.4		
5	12.4		
Fisher grade [14] (%)			
1	16.9		
2	14.6		
3	50.6		
4	18.0		
Rebleed prior to repair (%)	5.6	7.9	0.550
Vasospasm at arrival (%)	1.1	4.5	0.174
Time ictus to arrival (hours)	6.9 (2.8, 25.1)	6.9(2.8, 22.4)	0.968
Vasospasm (CTA-TCD) (%)			
None	*68.2*	*47.2*	*0.005*
Up to moderate in 1 vessel	12.9	14.6	0.750
Up to moderate in multiple vessels	14.1	16.9	0.618
Severe in 1 vessel	*2.2*	*12.4*	*0.010*
Severe in multiple vessels	2.2	9.0	0.051
Severe in 1 or more vessels	*4.5*	*21.4*	< *0.001*
Symptomatic vasospasm (%)	*9.0*	*21.4*	*0.022*
Treated with i.a. nimodipine (%)	2.2	7.9	0.087
Radiological DCI (%)	12.4	14.6	0.661
Length of stay (days)	*13.8* ± *8.3*	*16.5* ± *7.5*	*0.008*
Modified Rankin score [3] (%)			
0	24.3	17.7	0.316
1	44.6	34.2	0.187
2	18.9	24.1	0.441
3	4.1	6.3	0.528
4	6.8	13.9	0.148
5	1.4	3.8	0.343
Good outcome (grades 0–2, %)	91.9	82.3	0.078
Glasgow outcome scale extended [59] (%)			
8	29.7	19.0	0.121
7	40.5	38.0	0.745
6	18.9	16.5	0.690
5	4.1	8.9	0.229
4	5.4	10.1	0.278
3	1.4	6.3	0.113
2	-	1.3	0.332
Good outcome (grades 8–6, %)	*89.2*	*73.4*	*0.013*
30-day mortality (%)	11.2	7.9	0.444
1-year mortality (%)	15.7	11.2	0.380
Fatigue severity scale [28]			
Mean score	*4.11* ± *1.76*	*4.96* ± *1.39*	*0.018*
Mean score ≥ 4.00 (%)	53.8	68.2	0.180

Significant differences in italics

*i.a.* intra-arterial, *DCI* delayed cerebral ischemia, *TCD* transcranial Doppler ultrasonography, *CTA* computed tomography angiography, *WFNS* World Federation of Neurosurgery Societies [45]

### Outcome

The median time for follow-up was 5.3 months. There were no significant differences in 30-day or 1-year mortality, or in mRS, between the two groups (Table 5). Statin use was not a predictor of 1-year mortality, in contrast to high grade aSAH (OR 3.546 [95% CI 2.203–5.708], *p* < 0.001), hypertension (OR 1.772 [95% CI 1.100–2.856], *p* = 0.019), age (OR 1.073 [95% CI 1.050–1.096], *p* < 0.001), and female gender (OR 0.423 [95% CI 0.259–0.691], *p* < 0.001).

**Table 5:** Outcome in statin users and non-statin users

	Statin user	Non-statin user	*p*-value
Modified Rankin score [3] (%)			
0	21.7	15.6	0.065
1	41.4	47.9	0.155
2	19.1	20.3	0.730
3	8.6	5.0	0.097
4	8.6	9.2	0.792
5	0.6	2.0	0.335
Glasgow outcome score extended [59] (%)			
8	24.4	18.8	0.117
7	*36.9*	*47.1*	*0.020*
6	17.5	16.9	0.869
5	7.5	4.7	0.160
4	7.5	6.2	0.556
3	0.6	3.9	0.380
2	0.0	0.7	0.299
Fatigue severity scale [28] (mean score, SD)	4.480 ± 1.693	4.636 ± 1.683	0.493
Fatigue severity scale mean score ≥ 4 (%)	67.6	67.4	0.975
30-day mortality (%)	19.5	14.8	0.141
1-year mortality (%)	24.5	19.3	0.142

Significant differences in italics. Follow-up—median 5.3 months IQR (4.2, 7.9)

*SD* standard deviation

**Fig. 2:**
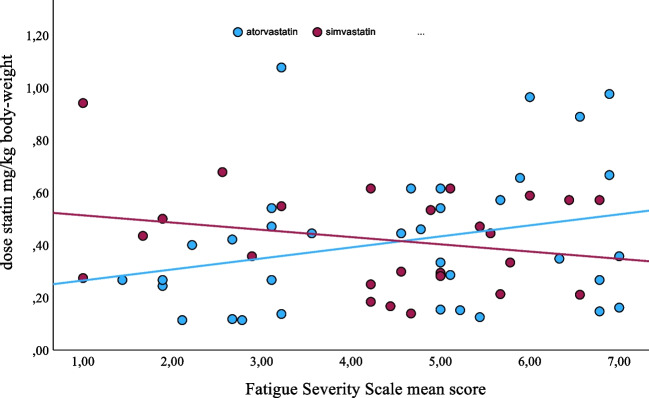
Dose (mg/kg body weight) of atorvastatin (blue dots) and simvastatin (red dots) versus fatigue severity scale [28] mean score

More non-statin users survived to GOSE grade 7 (Table 5). This difference was no longer observed in the matched pair analysis; on the contrary, we found more statin users surviving to a favorable outcome in terms of GOSE 8–6 (Table 4). There was no significant difference in levels of fatigue on group level, whereas statin users had a lower FSS mean score in the matched pair analysis (Tables 4 and 5). The FSS mean score was not related to the statin dose (Fig. 2). Still, the frequency of clinically significant fatigue (FSS mean score ≥ 4.00) was not statistically significant different in statin users (53.8%) and non-statin users (68.2%).

## Discussion

The core finding of the present study was that patients with pre-ictal use of statins had a reduced frequency of severe radiological/sonological VS, symptomatic VS, and length of stay. Statin users had more often a favorable outcome in terms of GOSE 8–6 and lower levels of fatigue, whereas mortality was similar to that of non-statin users.

### Vasospasm

Radiological/sonological VS was presently less frequent in statin users. They being more than a decade older than their non-user counterparts may have contributed to this finding. Whether age is protective against VS is though controversial. Ryttlefors et al. [47] found that age was not a predictor for radiological/sonological VS or DIND; in contrast, Frontera et al. [17] found that younger age enhanced the risk of developing radiological VS. Yin et al. [60] concluded that both age and modified Fisher were positive predictors of symptomatic VS. Our findings concur with this as higher age presently was an independent protective predictor of radiological/sonological and symptomatic VS while larger amounts of subarachnoid blood (Fisher grades 3 and 4) more than tripled the risk to develop VS. Notwithstanding extensive research, the exact pathophysiological mechanism behind the development of VS remains unknown. There seems to be some consensus that there is a cascade of events in the breakdown of hemoglobin that leads to inflammation causing thickening of the arterial wall and thereby vessel lumen narrowing. With more blood, this reaction and consequently development of VS is assumed to be more pronounced [14]. The Fisher score takes account of the amount of subarachnoid blood as well as ICH and IVH, and finding Fisher grade to be an independent predictor of VS supports the abovementioned pathophysiological notion.

Vessel narrowing documented radiological/or by TCD may be a different entity than symptomatic VS which is an exclusion diagnosis of neurologic decline. Radiological/sonological and symptomatic VS do not always concur; i.e., severe narrowing of arteries can be observed in the absence of neurologic symptoms and vice versa [7]. Symptomatic VS may be caused by narrowing of very small arteries or even due to microcirculatory disturbances [42]. This would only be diagnosed by perfusion studies. Furthermore, neither large vessel narrowing nor symptomatic VS necessarily translates into DCI. Symptomatic VS/clinical DCI and/or radiologically established DCI seems to be the result of more complex and multifactorial processes than mere vessel narrowing [21]. The array of contributing factors is wide and includes early brain injury [18], cortical spreading depression [10], autoregulatory failure [5], delayed apoptosis [61], blood–brain barrier disruption [23], microthromboembolism [57], oxidative stress [1], and inflammation [44]. The pleiotropic neuroprotective properties of statins include increased neurogenesis and synaptogenesis, increased release of neurotrophic factors, reduction of oxidative stress, and inhibition of inflammation both central and peripheral [54]. Statins enhance the conversion of plasminogen to plasmin, which breaks down the fibrin clot, and they inhibit prothrombin formation and platelet function, thereby counteracting microthromboembolism [54]. A review by Vaughan and Delanty [55] concluded that statins exert neuroprotective effects by attenuating the effects of ischemia on the brain vasculature and parenchyma. They found that statins’ neuroprotective effect comes from an upregulation of eNOS, along with reducing oxidation and modulation of the inflammatory response. In other words, statins interfere with many of the mechanisms participating in the phenomenon of symptomatic VS and DCI. From this, it is not surprising that we found a reduction in symptomatic VS both on group level and in the case–control design in the statin users.

The extent to which symptomatic VS reaches the endpoint of manifest DCI will vary with institutional treatment algorithms. We used repeated i.a. nimodipine application as a rescue therapy for severe and/or symptomatic VS, whereas other departments may choose different treatments like balloon angioplasty or induced hypertension and volume management alone [15, 26]. This would impact the constellation of symptomatic VS and manifest DCI in the respective neurosurgical centers and hence the results reported in the literature. Furthermore, all our patients received i.v. or peroral VS prophylaxis with nimodipine for 21 days after the ictus. I.v. and peroral nimodipine does not reduce vessel narrowing, but it reduces DCI and improves outcome [12]. Our prophylactic regime may have had an impact on the frequency of VS, DCI, and outcome. Guidelines still recommend merely the prophylactic use of peroral nimodipine [22], and i.v. nimodipine is not even available in some countries like for instance Northern America. Differences in VS prophylaxis and rescue treatment may therefore be a reason to diverging results regarding the effect of statins on VS and outcome in the literature.

Tseng et al. [53] randomized 40 aSAH patients to 14 days of treatment with 40 mg pravastatin within 72 h after the ictus and found reduced VS, shortened duration of impaired autoregulation, and decreased mortality as compared to 40 aSAH patients treated with placebo. Pravastatin is hydrophilic and does not pass the intact blood–brain barrier. In aSAH, however, the blood–brain barrier may be disrupted, allowing the pleiotropic effects of pravastatin taking action in the brain. They defined VS as TCD velocities in excess of 120 cm/s and a Lindegaard ratio greater than 3 [34, 53]. In contrast, other statin studies defined VS as velocities > 200 cm/s [6], ≥ 160 cm/s [19], or by cerebral angiography [8, 37, 38, 41]. Furthermore, most studies insonated only the MCA, with exception of Vergouwen et al. who also measured velocities in the ACA [56]. This heterogeneity may contribute largely to the diverging findings among the studies performed on the effect of statins in aSAH patients. We presently scored VS from both CTA and TCD findings investigating the MCA, ACA, PCA, and distal extracranial ICA and considered the grade of vessel narrowing using the Lindegaard ratio, not the absolute velocities. We further used the highest scores of either method. Our radiological/sonological VS findings may hence differ from that of others but may also be more robust.

### Outcome

The largest multicenter RTC, the STASH study [27], did not find any benefit in terms of mortality or functional outcome from 21 days of simvastatin use starting upon arrival. It is not clear after how long time of use the pleiotropic effect of statins may take effect; in fact, it has been shown that only long-term use (> 6 months) reduces cholesterol levels in the CSF [54]. This may be a contributing factor in discrepancies between retrospective statin studies and RCTs. Lizza et al. [36] studied pre-admission use of statins and did not find a reduction in VS or better outcome. However, many of their patients discontinued their own statin dose after admission for aSAH. Parra et al. [43] found significantly better outcome, reduced DCI, and that statins prevented the highest TCD velocities among those using statins at the time of hemorrhage. Their study, however, included only few patients, all of them older than 65 years. It is not clear if the RTCs corrected for patients with pre-ictal statin use when allocating them to a treatment arm. Potentially, to not exclude those on prior statin use could have an impact on the results. Apart from non-statin users surviving more often to a mRS of 4, we found similar results for mortality and functional outcome in our two groups. This needs to be interpreted in light of our statin users being older, more often male, and having more often hypertension which are predictors of mortality and poor outcome in the present study as well as in literature [30, 46]. It is hence not surprising that we actually found better functional outcome in our statin users in the matched pair analysis where we corrected for the imbalance in age, gender, and hypertension.

An important factor in scoring functional outcome after aSAH is the presence of post-aSAH fatigue. Given the anti-inflammatory properties of statins, one could anticipate that the levels of fatigue differ between statin users and non-statin users. Also, reduced GCS at ictus and severe vasospasm were found to be independent predictors of post-aSAH fatigue [58] indicating a link between VS and fatigue. We did not find any difference in levels of fatigue on group level, whereas FSS mean scores were lower in statin users in the matched pair analysis. This indicates that the pathophysiology of fatigue is more complex than inflammation alone and/or that there are other determinants decisive for the development of chronic post-aSAH fatigue. Possibly, hypertension may be such a determinant.

Finally, not only the type of statin but also its dose could be important for its effect on VS and outcome. A meta-analysis found weak evidence that a higher total statin dose reduced the risk for VS, DIND, and mortality [52]. They reported heterogeneity among the individual studies and suggested a better dosing strategy in future RCTs. Analyzing data in relation to dose/kg body weight reduces the aspects of pharmacokinetic influence, and our study is the first to include this facet.

### Strengths and limitations

The retrospective, single-center nature of this study limits the external validity of our findings. Likewise will institutional treatment algorithms differ and contribute to this limitation. On the other hand, our data were collected systematically and continuously in a quality register assuring a better quality than a strictly retrospective data acquisition. All patients were treated in accordance with our institutional guideline, reducing bias from individual neurosurgeons treatment preferences. Our guidelines also assured that statin users continued their medication after admission. The number of included patients in our study is considerably higher than in previous studies but may still be too low to render significant differences in the defined primary outcome. Our fraction of statin users (21.2%) is higher than in previous reports, which renders the group-wise comparison more robust and enabled us to match a larger number of pairs. We do not know the length of pre-ictal statin use; however, it is uncertain which role this plays. Furthermore, we cannot eliminate the possibility of bias by unmeasured confounders. For instance, statin users may be more health-conscious and seek/follow medical advice more wholeheartedly than non-statin users. On the other hand, statin users may have had a higher (cardiac) co-morbidity not accounted for in this study but with possible impact on outcome.

We used clear definitions of VS and investigated both radiological/sonological and symptomatic VS. This is a strength but also hinders comparability with other studies. Scoring symptomatic VS and DCI is prone to individual interpretation, and there is a risk of underreporting. We assume this risk to be similar in our cohort and those of other studies.

Most of our statin users had prescribed the lipophilic atorvastatin that has been studied little in the context of aSAH, so that our study provides data regarding this type of statin. Many other studies investigated the effect of the hydrophilic statin pravastatin, whereas only 2.5% of our statin users were administered this drug. Radiological DCI was mainly diagnosed with CT scans, and exclusive use of magnetic resonance imaging would have been more sensitive, so that our DCI frequency may be lower than the real number. This would, however, affect both our two groups equally.

## Conclusions

Patients with pre-ictal and continued use of statins have a reduced occurrence of severe radiological/sonological VS, symptomatic VS, shorter length of stay, and more often favorable outcome in terms of GOSE 8–6 and levels of fatigue. Mortality is similar in statin users and non-statin users. Even though larger multicenter studies with common, strict protocols for prevention, diagnosis, and treatment of vasospasm are needed to finally establish the value of statins in aSAH, continuation of pre-ictal statin use seems worthwhile.

## Data Availability

Data from the present study can be made available upon reasonable request.

## Abbreviations

ACA: Anterior cerebral artery
ACoA: Anterior communicating artery
aSAH: Aneurysmal subarachnoid hemorrhage
ASDH: Acute subdural hematoma
CT: Computed tomography
CTA: Computed tomographic angiography
CPP: Cerebral perfusion pressure
CSF: Cerebrospinal fluid
DCI: Delayed cerebral ischemia
DIND: Delayed ischemic neurologic deficit
FSS: Fatigue severity scale
GOSE: Glasgow outcome score extended
i.a.: Intra-arterial
ICA: Internal carotid artery
ICH: Intracerebral hemorrhage
IVH: Intraventricular hemorrhage
MCA: Middle cerebral artery
MRI: Magnetic resonance imaging
mRS: Modified Rankin score
NO: Nitrogen oxide
PCA: Posterior cerebral artery
RCT: Randomized controlled trial
TCD: Transcranial Doppler ultrasonography
VS: Vasospasm
WFNS: World Federation of Neurosurgical Societies
